# The one-stop-shop approach: Navigating lumbar 360-degree instrumentation in a single position

**DOI:** 10.3389/fsurg.2023.1152316

**Published:** 2023-03-16

**Authors:** Maximilian Schwendner, Raimunde Liang, Vicki M. Butenschön, Bernhard Meyer, Sebastian Ille, Sandro M. Krieg

**Affiliations:** ^1^ Department of Neurosurgery, School of Medicine, Technical University of Munich, Klinikum rechts der Isar, Munich, Germany; ^2^ TUM Neuroimaging Center, School of Medicine, Technical University of Munich, Klinikum rechts der Isar, Munich, Germany

**Keywords:** one-stop shop, spinal navigation, lateral instrumentation, navigated pedicle screw placement, pyogenic spondylodiscitis

## Abstract

**Objective:**

Treatment strategies of patients suffering from pyogenic spondylodiscitis are a controverse topic. Percutaneous dorsal instrumentation followed by surgical debridement and fusion of the infectious vertebral disc spaces is a common approach for surgical treatment. Technical advances enable spinal navigation for dorsal and lateral instrumentation. This report investigates combined navigated dorsal and lateral instrumentation in a single surgery and positioning for lumbar spondylodiscitis in a pilot series.

**Methods:**

Patients diagnosed with 1- or 2-level discitis were prospectively enrolled. To enable posterior navigated pedicle screw placement and lateral interbody fusion (LLIF) patients were positioned semi-prone in 45-degree fashion. For spinal referencing, a registration array was attached to the pelvic or spinal process. 3D scans were acquired intraoperatively for registration and implant control.

**Results:**

27 patients suffering from 1- or 2-level spondylodiscitis with a median ASA of 3 (1–4) and a mean BMI of 27.9 ± 4.9 kg/m^2^ were included. Mean duration of surgery was 146 ± 49 min. Mean blood loss was 367 ± 307 ml. A median of 4 (4–8) pedicle screws were placed for dorsal percutaneous instrumentation with an intraoperative revision rate of 4.0%. LLIF was performed on 31 levels with an intraoperative cage revision rate of 9.7%.

**Conclusions:**

Navigated lumbar dorsal and lateral instrumentation in a single operation and positioning is feasible and safe. It enables rapid 360-degree instrumentation in these critically ill patients and potentially reduces overall intraoperative radiation exposure for patient and staff. Compared to purely dorsal approaches it allows for optimal discectomy and fusion while overall incisions and wound size are minimized. Compared to prone LLIF procedures, semi-prone in 45-degree positioning allows for a steep learning curve due to minor changes of familiar anatomy.

## 1. Introduction

Pyogenic spondylodiscitis (PS) is a potentially life-threatening bacterial infection, that represents 3%–5% of all cases of osteomyelitis (1, 2). Incidence rates in Europe are reported ranging from 0.4 to 5.8 /100,000 persons every year, while data from the German Federal Statistical Office reported rates above 10/100,000 persons every year (1–3). Magnetic resonance imaging (MRI) remains the most important method of imaging for spondylodiscitis, while new imaging methods such as 18F-fluorodeoxiglucose positron emission tomography (FDG-PET) are frequently used in patients with contraindications for MRI, for the differentiation between spondylodiscitis and severe degenerative changes as well as to exclude metastatic infections (4–6).

Treatment strategies regarding PS vary widely. The primary indications for surgery include progressive neurological impairment, epidural abscess, pain caused by spinal instability, progressive deformity, or failure to respond to conservative treatment (7–10). However, in the last years there is an increasing trend towards surgical treatment of newly diagnosed PS as an initial treatment strategy due to early mobilization and reduced medical complications (11). Minimally invasive surgery (MIS) including percutaneous pedicle screw placement was shown to provide superior surgical outcomes such as significantly shorter durations of surgery, a lower perioperative need for blood products and a shorter hospital stays for multiple indications including pyogenic spondylodiscitis, traumatic fractures and spinal metastases (12–14).

At our institution surgical treatment of PS of the thoracolumbar spine is mostly performed *via* dorsal instrumentation by percutaneous pedicle screw placement and debridement of the disc space and cage placement *via* lateral lumbar interbody fusion (LLIF) in a second surgery. A surgical approach to perform dorsal and lateral instrumentation in one single positioning surgery under fluoroscopic control was reported by Drazin et al. in 2015 in cohort of patients with degenerative spinal disease (15).

With this study we report our initial experience with a combined navigated 360-degree instrumentation by dorsal percutaneous pedicle screw placement and LLIF procedure in a semi-prone 45-degree position. Clinical and radiological outcome of patients diagnosed with single or two-level spondylodiscitis of the lumbar spine treated are analyzed.

## 2. Methods

### 2.1. Hypothesis

Our hypothesis is that combined 360-degree instrumentation by dorsal percutaneous pedicle screw placement and debridement of the intervertebral disc space and cage placement *via* LLIF performed in a single positioning under guidance of spinal navigation offers a safe and accurate treatment strategy for the surgical treatment of patients suffering from PS of the lumbar spine. Due to the quick and straightforward procedure including extensive discectomy, multimorbid PS patients can be treated safe and without recurrence.

### 2.2. Ethics

The study was approved by the local ethics board (registration number: 2022-22_1-S-KH). We performed the study in accordance with the Declaration of Helsinki and in accordance to the STROBE statement.

### 2.3. Study protocol

Patients diagnosed with single- or two-level PS of the lumbar spine who were surgically treated by a combined navigated lumbar 360-degree instrumentation in a single positioning at our institution. between June 2021 and October 2022 were prospectively enrolled. Subsequently, patients undergoing conservative treatment or instrumentation in a staged approached were excluded. Perioperative complications as well as the clinical and radiographic outcome were further analyzed.

### 2.4. Indication for surgery

All patients underwent preoperative MRI and CT-imaging to diagnose PS. Indication for surgery was an infectious alteration of the intervertebral discs and anterior longitudinal ligament combined with a bony destruction of the adjacent vertebral body endplates. Patients suffering from low-back pain, sciatica or neurological deficits were scheduled for surgical treatment.

### 2.5. Spinal navigation

For spinal navigation, a mobile navigation system (Medtronic Stealth™ S7, Medtronic, Minneapolis, Minnesota, USA) or operating room-installed ceiling-mounted navigation system (Curve, Brainlab, Munich, Germany) was used in all cases. For the registration of the spinal navigation system, an intraoperative 3D-imaging scan using a mobile cone-beam computed tomography scanner (CT) (O-arm II, Medtronic, Minneapolis, Minnesota, USA) or operating room-installed CT (Brilliance CT Big Bore, Philips, Amsterdam, Netherlands) covering all planned vertebral bodies was performed accordingly. For referencing during surgery, a registration array equipped with reflective sphere markers was attached to a pelvic or spinal process.

### 2.6. Surgical procedure

**Figure 1:**
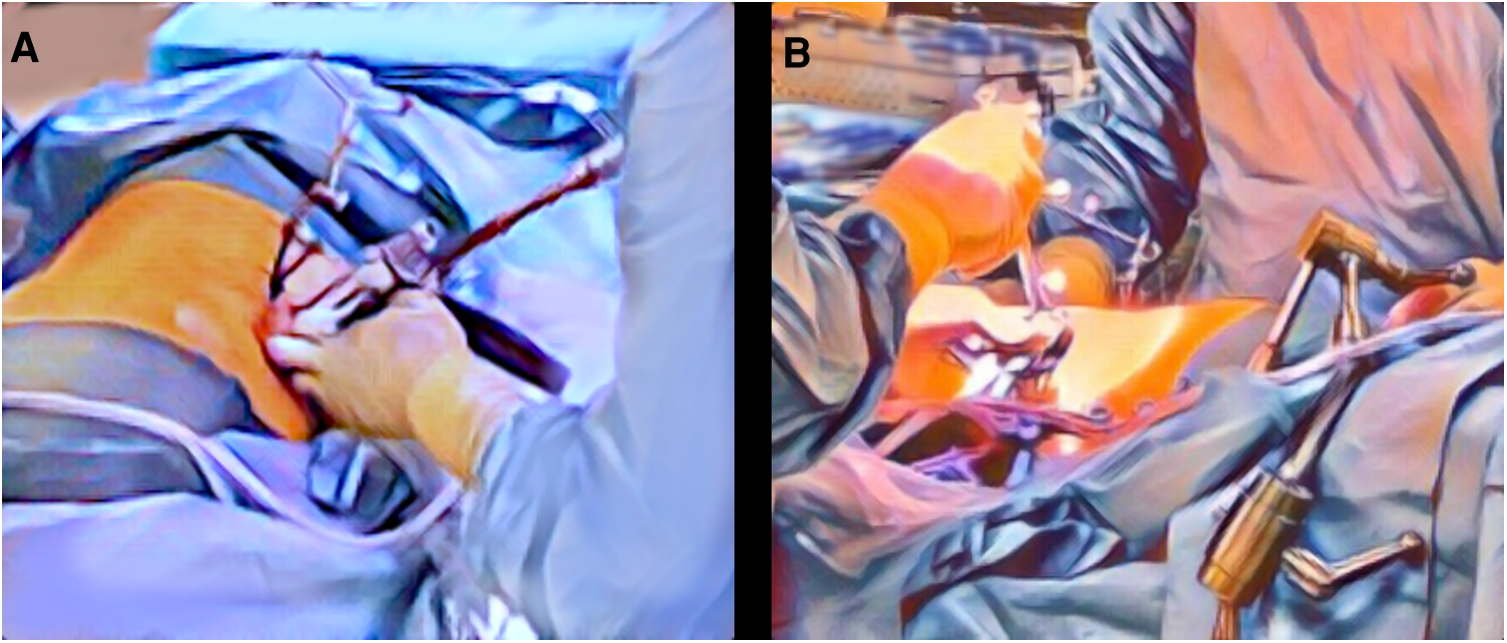
Patient positioning and setup. The patient is positioned semi-prone in a 45-degree right-sided positioning to enable sufficient surgical access for both surgical steps—navigated pedicle screw placement (**A**) and lateral discectomy and fusion (**B**). For referencing during surgery, a registration array is attached to a pelvic or spinal process. The surgical sites for pedicle screw placement (**A**) and lateral interbody fusion (**B**) are illustrated.

The patient was positioned semi-prone in a 45-degree right-sided positioning on the operation table during the whole procedure (Figure 1). For referencing during surgery, a registration array was attached to a pelvic or spinal process. For the registration of the navigation system, an intraoperative 3D-imaging scan covering all planned vertebral bodies was performed. The first surgical step was bilateral dorsal instrumentation by navigated percutaneous pedicle screw placement (CD Horizon Solera, Medtronic, Minneapolis, Minnesota, USA) (Figure 1) using a navigated drill-guide and Kirschner wires. Starting the procedure with pedicle screw placement is crucial for an optimal accuracy of the spinal navigation. Next, lateral instrumentation was performed using a left-sided retroperitoneal approach by a tubular retractor (Clydesdale Spinal System, Medtronic, Minneapolis, Minnesota, USA) (Figure 1). In terms of total navigation, this procedure was performed under guidance of the spinal navigation as well, allowing for constant image referencing during all surgical steps (skin incision, preparation, implant placement). For planning of the skin incision and surgical preparation a navigated pointer was used. The infectious vertebral discs were resected using a navigated chisel, and anterior decompression was performed in case of spinal canal stenosis. Titanium cages (Juliet LL, Spineart, Plan-Les-Ouates, Switzerland) prepared with topic gentamicin or vancomycin were implanted into the disc spaces. Positioning was reviewed using the navigated pointer. After completing cage placement, a second intraoperative 3D imaging set including updated spinal navigation was performed for implant control. In case of implant revision, a second control scan was acquired. Afterwards, rods were implanted bilaterally and a stepwise wound closure was performed.

### 2.7. Radiographic analysis

**Figure 2:**
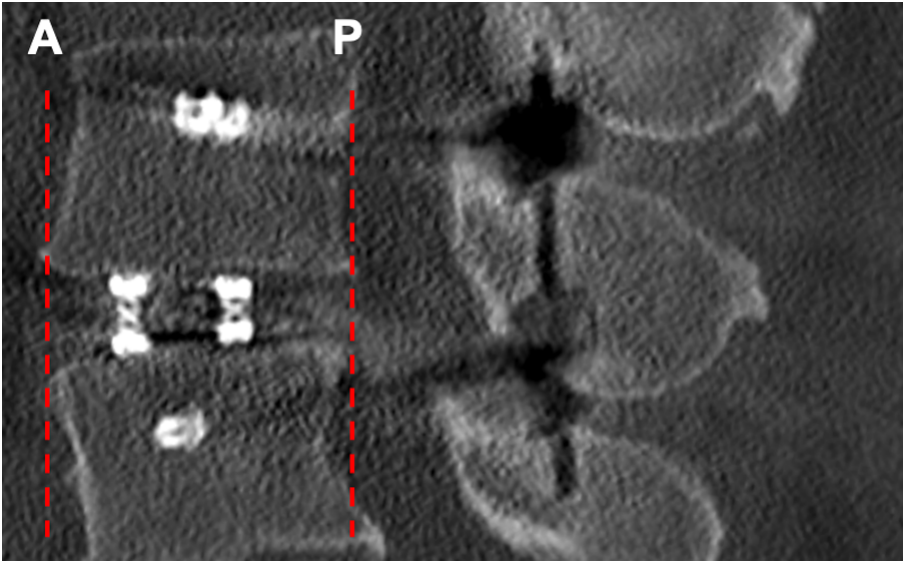
Radiographic evaluation of cage placement. Figure 2 shows an intraoperative CT-scan after 360-degree instrumentation of the lumbar spine in a sagittal view. Cage placement was evaluated in anterior (**A**) and posterior (***P***) orientation in relation to the limits of the upper and lower endplate of the adjacent vertebrae.

For radiographic analysis, intraoperative 3D-imaging and postoperative imaging was analyzed. Pedicle screw positioning was evaluated on intraoperative 3D-scans according to the Gertzbein and Robbins scale (GRS) (16). Cage placement was evaluated in relation to the center of the intervertebral disc space and limits of the upper and lower endplate of the adjacent vertebral bodies (Figure 2).

### 2.8. Data analysis

Statistical analyses were performed using Prism (version 9.1.1, GraphPad Software, La Jolla, CA, USA). Descriptive statistics including mean, median, minimum, maximum, and standard deviation were calculated for patient characteristics as well as radiographic measurements. For statistical testing between groups, Mann–Whitney U tests for unpaired samples as well as Fisher's exact tests with a level of significance set at *p* < 0.05 were performed. Figures were created using Prism (version 9.1.1, GraphPad Software, La Jolla, CA, USA).

## 3. Results

### 3.1. Patient characteristics

A consecutive series of 27 patients (8 women, 19 men) with 32 levels of spondylodiscitis of the lumbar spine was analyzed (Table 1). All patients underwent 360-degree instrumentation of the lumbar spine in a single surgery. No access surgeon was used. Mean age was 68.5 ± 13.7 (31.9–86.4) years, with 20 patients (74.1%) aged 65 years or above. Mean BMI was 27.9 ± 4.9 (21.3–38.7) and the median ASA-PS (American Society of Anaesthesiologists physical status) class was 3 (1–4) (Table 1). One patient showed a hip flexor weakness preoperatively due to a psoas abscess, while all other patients showed no sensory or motor deficit related to the spinal infection.

**Table 1:** Patient data.

*n* (%)	
Number of patients	27 (100)
Female gender	8 (29.6)
Age at surgery (year; mean ± SD; range)	68.5 ± 13.7 (31.9–86.4)
• Age < 65 year	7 (25.9)
• Age ≥ 65 year	20 (74.1)
BMI (mean ± SD; range)	27.9 ± 4.9 (21.3–38.7)
**ASA-PS class**	
Median (range)	3 (1–4)
• Class 1 • Class 2 • Class 3 • Class 4	4 (14.8) 6 (22.2) 16 (59.3) 1 (3.7)

Table 1 illustrates patient data including body mass index (BMI) and ASA-PS (American society of anaesthesiologists physical status) class.

### 3.2. Surgical data

**Figure 3:**
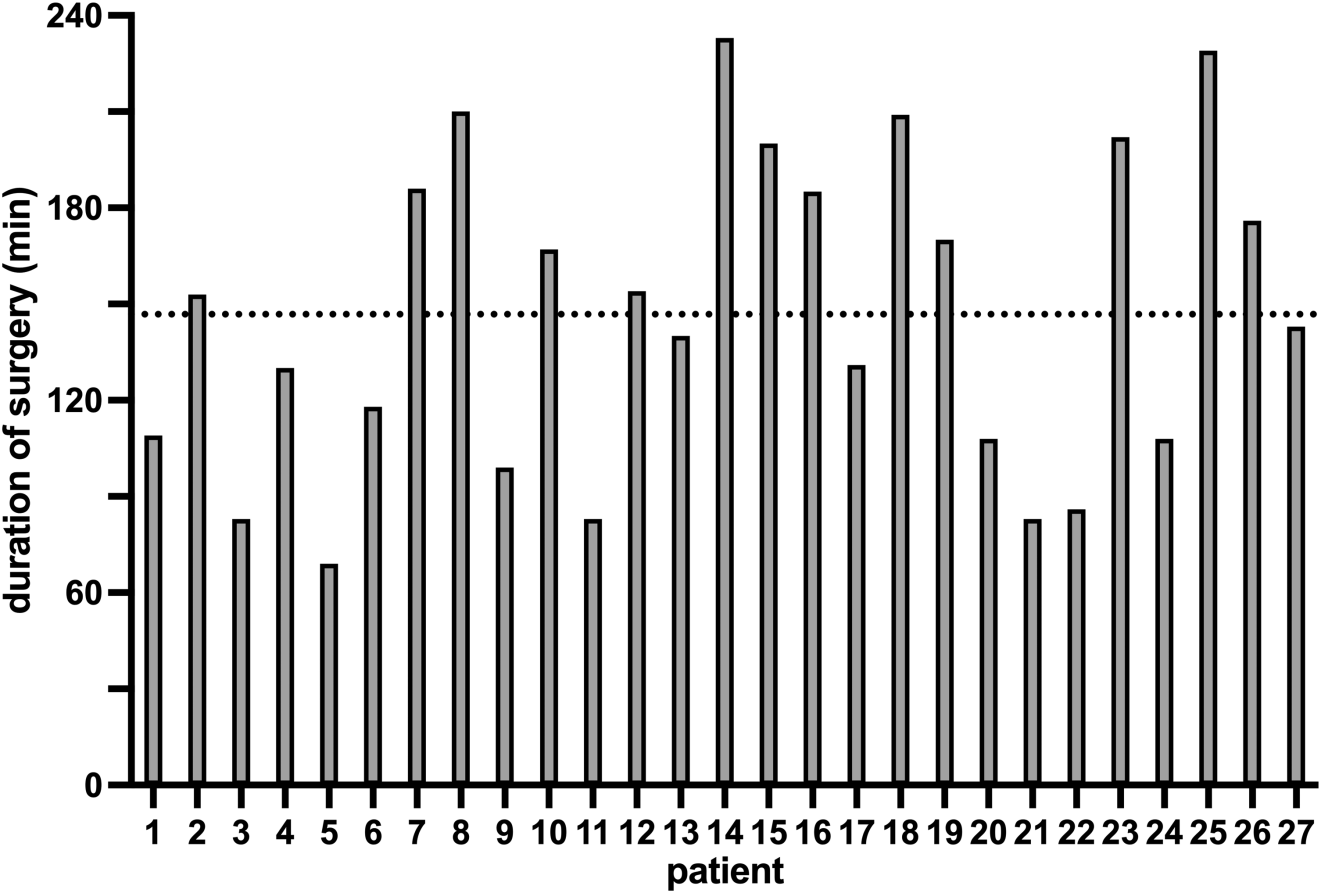
Duration of surgery. The duration of surgery of all 27 patients with an average duration of 147 min (ticked line), a minimum duration of 69 min (patient 5) and a maximum duration of 233 min (patient 14) is illustrated in this figure.

Duration of surgery was 146 ± 49 min (69–233) minutes (Figure 3). Estimated intraoperative blood loss was 367 ± 307 (50–1300) ml.

Regarding dorsal instrumentation, 124 screws were implanted in total. In 20 patients, single segment instrumentation was performed, while in five (18.5%) patients two segments and in two (7.4%) patients three segments were instrumented (Table 2). Overall, five (4.0%) pedicle screws were revised in two patients, in one case an intraoperative revision of two screws and a postoperative revision of another screw was performed.

**Table 2:** Surgical data.

*n* (%)	
Levels of LLIF (median; range)	1 (1–2)
Vertebrae of PS instrumentation (median; range)	2 (2–4)
Percutaneous dorsal instrumentation	27 (100.0)
**Levels of Spondylodiscitis**	
• L 1/2 • L 2/3 • L 3/4 • L 4/5	3 (9.4) 9 (28.1) 9 (28.1) 11 (34.4)

This table illustrates the surgical procedures regarding spinal levels treated by dorsal instrumentation and lateral instrumentation.

Regarding lateral instrumentation, 30 cages including one expandable cage due to severe end plate osteolysis (Obelisc, Ulrich Medical, Ulm, Germany) were implanted. Intraoperative cage revision was performed in three cases, and in two cases the cage was further inserted in the intervertebral disc space after the control scan. In one case no cage was implanted as reduced height of the intervertebral disc space did not allow for cage placement.

### 3.3. Radiological outcome

**Figure 4:**
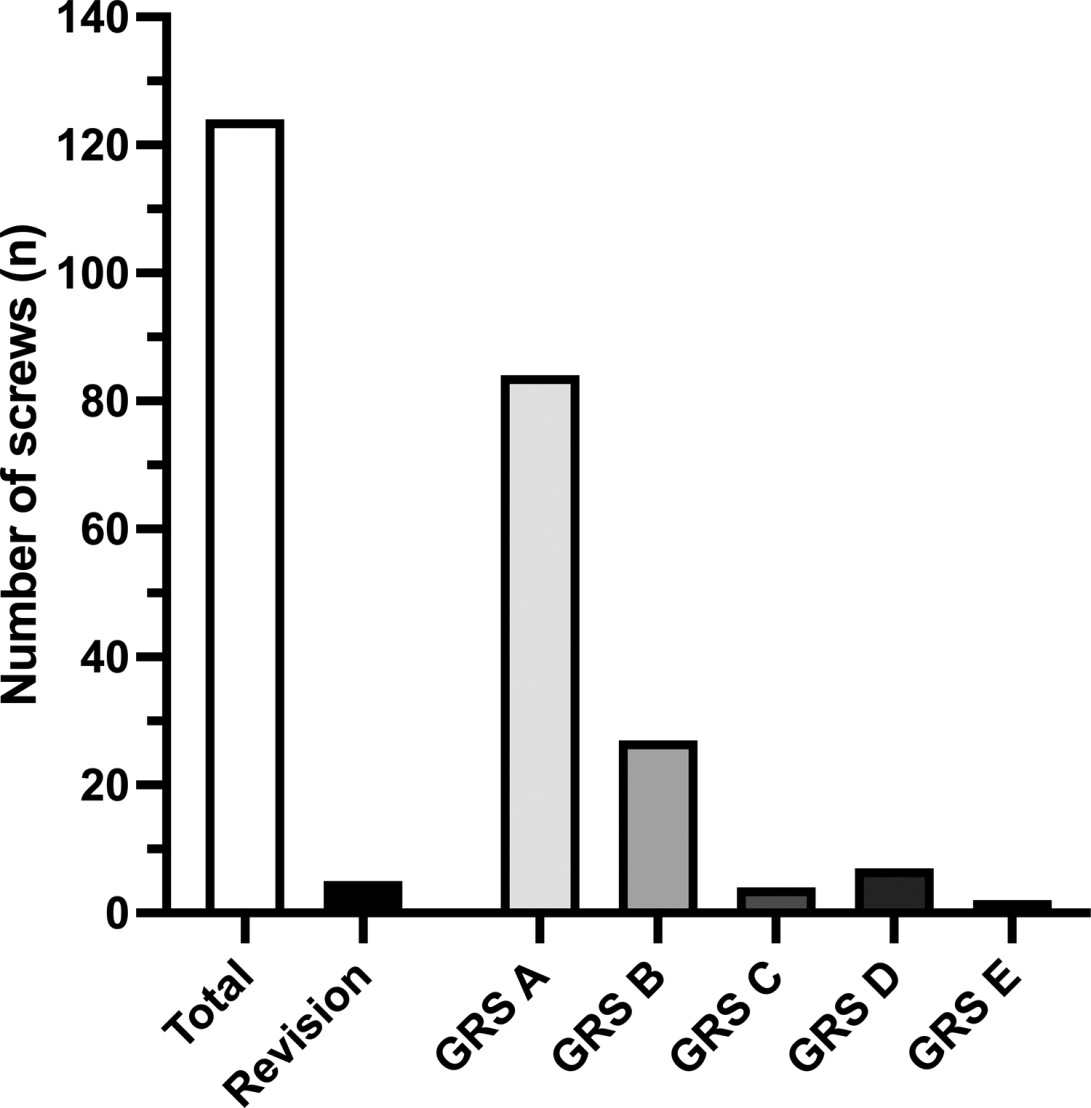
Accuracy of pedicle screw placement. Figure 4 illustrates the accuracy of pedicle screw placement regarding the radiographic evaluation according to the Gertzbein and Robbins scale (GRS) and revision rates of the pedicle screws.

Pedicle screw placement was reviewed on initial intraoperative imaging instrumentation according to GRS. Screw positioning rated GRS A and B was achieved in 111 screws (89.5%) (Figure 4).

Regarding cage placement in sagittal plane—cages showed contact to the anterior border of the upper and lower endplates of the adjacent vertebral bodies in nine (30.0%) cases with the cage exceeding the endplates in two cases (6.7%), and contact to the posterior border in two (6.7%) cases (Figure 2).

### 3.4. Clinical outcome

All patients were treated with intravenous antibiotics for a minimum of 14 d, followed by 10 weeks of oral antibiosis. Treatment schemes were individually adapted and discussed during interdisciplinary ward rounds lead by the department of microbiology. Three patients (11.1%) required ICU treatment, with a mean ICU stay of 13.3 (3–28) days. Mean hospital stay was 18.5 ± 12.2 (4–64) days, and 11 patients were referred to a secondary care hospital for further intravenous antibiosis and rehabilitation.

Regarding surgery-related neurological deficits persisting at the time of discharge one patient showed postoperative deterioration of motor function related to an intraoperative nerve-root injury. Atrophic wound healing disorders requiring local surgical wound revision occurred in five cases (18.5%) with one patient aged under 65 years (14.2%) and four patients (20%) over 65 years.

Follow-up examinations were available on 25 patients on 129 ± 111 (22–484) d postoperatively. One patient had to undergo a second surgery due to ongoing bony destruction of the vertebral body leading to cage subsidence requiring a complete vertebral body replacement using an expandable cage. Two multimorbid patients died due to cardiac complications induced by the systemic infection. Another patient suffered a severe acute respiratory syndrome coronavirus 2-infection four weeks postoperatively and was subsequently treated with best supportive care due to multiple comorbidities and advanced age. Two patients had to be re-hospitalized due to persistent infection parameters in blood testing. Further diagnostics ruled out persistent spondylodiscitis—both patients were effectively treated by a prolongated scheme of intravenous antibiotics followed by oral antibiosis. All six patients younger than 65 years with follow-up data available showed regular healing.

## 4. Discussion

### 4.1. Surgical treatment in pyogenic spondylodiscitis

Treatment strategies regarding PS vary widely amongst centers worldwide. However, in the last years there is an increasing trend towards surgical treatment of newly diagnosed spondylodiscitis as an initial treatment strategy (17–19). Regarding surgical treatment strategies of PS of the thoracolumbar spine, dorsal percutaneous pedicle screw instrumentation with additional surgical debridement of the intervertebral disc space *via* posterior discectomy or LLIF including cage implantation has been proven to show most favorable outcomes, especially regarding long-term fusion rates (17, 20–22). For the choice of implants, titanium implants should be favored over polyetheretherketone (PEEK) as higher rates of implant loosening were reported for PEEK in patients treated for spondylodiscitis (22–24). A significant rate of postoperative wound healing disorders of 18.5%, which was similar in patients below and over the age of 65 years, was observed in this study. In previous studies, rates of wound healing disorders greatly vary between 2.4% and 12.3% (20, 22). The definition of a wound healing disorder might vary between studies—in this study all patients requiring surgical revision under general and local anesthesia were considered.

### 4.2. Single positioning surgery

Most patients diagnosed with PS are of higher age and suffer multiple comorbidities. Therefore, the total duration of surgery and blood loss should be kept as low as possible, intraoperative stress minimized and early mobilization and rehabilitation should be facilitated. A study by Tong et al. in 2019 successfully combined dorsal and lateral instrumentation in a single surgery with an intraoperative switch from prone positioning to lateral positioning in patients with single-level PS (25). In terms of intraoperative patient positioning in general, the conventional prone position bears the risk of cardiovascular complications including cardiac arrest as well as the risk of hypovolemia, reduced pulmonary compliance, and postoperative vision loss (26, 27). A lateral positioning helps to partially reduce these complications. Furthermore, combining lateral and dorsal instrumentation in one single positioning helps to reduce the overall time of the patient under general anesthesia, especially when compared to two separate surgeries but also compared to two patient positionings in one surgery (28). A large meta-analysis published by Mills et al. in 2021 compared single position lumbar fusion surgery in both lateral and prone positioning for patients with lumbar degenerative disease, spondylolisthesis, or radiculopathy to surgery in a staged approach, demonstrating overall significantly reduced durations of surgery and a reduced length of hospitalization (29). Nevertheless, higher complication rates regarding pedicle screw placement in a lateral positioning were reported for this approach (29).

### 4.3. Spinal navigation

Navigated pedicle screw placement has been proven to be advantageous over conventional screw placement, especially when performing percutaneous transmuscular instrumentation (30, 31). Though, applying spinal navigation has proved to be advantageous also in lateral instrumentation in terms of constant image referencing during all surgical steps (skin incision, preparation, implant placement) and for minimizing radiation exposure to the staff. In patients with PS, debridement of the disc space and cage placement is performed to reduce bacterial load quickly and to facilitate fusion without the intention to perform major corrections by multi-level instrumentation. Otherwise, the accuracy of spinal navigation might be impaired during cage placement.

When applying spinal navigation for both—dorsal and lateral instrumentation—another registration scan is warranted when repositioning the patient. Sellin et al. applied CT-guided navigation for simultaneous lateral interbody fusion and pedicle screw placement in lateral positioning showing reduced radiation exposure and reduced duration of surgery, especially as intraoperative navigation replaced intraoperative fluoroscopy and therefore allowed the parallel execution of both procedures (32). Ikuma et al. compared single-positioning surgery in a right lateral decubitus positioning for cases with and without spinal navigation, showing a significantly reduced duration of surgery for cases with spinal navigation as spinal navigation partially enabled simultaneous anterior and posterior instrumentation (33).

Regarding the application of robot-assisted navigated pedicle screw placement for 360-degree surgeries in a single positioning, Sinkov et al. reported limitations regarding contralateral pedicle screw placement due to problems accessing the surgical site in lateral decubitus positioning (34).

### 4.4. 45-degree semi-prone patient positioning

In our study, patients were positioned semi-prone in a 45-degree right-sided positioning on the operation table during the whole procedure (Figure 1). Compared to conventional 90°-lateral decubitus positioning, the accessibility of the surgical site especially for pedicle screw placement is improved. Dorsal and lateral instrumentation were performed under spinal navigation to enable continuous 3D visualization during all surgical steps, which helps to adapt to the modified patient positioning. In our experience, semi-prone 45-degree positioning allows for a steep learning curve due to minor changes of familiar anatomy, especially compared to prone LLIF and procedures like pedicle screw placement in a lateral decubitus position. When compared to purely dorsal approaches, this combined surgical approach allows for optimal discectomy and fusion. In addition, overall incisions and wound sizes are minimized by MIS, as percutaneous pedicle screw placement can be performed without the necessity of additional dorsal decompression. The spinal canal remains untouched.

Regarding the accuracy of pedicle screw placement in our study, two patients (7.4%) were reported with pedicle screw revision in this study, resulting in a total screw revision rate of 4.0%, which is comparable to previous studies in patient positioning in prone position (35, 36).

### 4.5. Limitations

This study was performed as a pilot study to confirm the feasibility and safety of this surgical approach. Therefore, no control group was included. In this study, patients with single or two-level PS of the lumbar spine were treated. In more extensive cases of PS including patients requiring multi-level instrumentation or instrumentation of the middle and upper thoracic spine a staged approach should be favored. When applying a modified patient positioning, the potential time benefit should be weighed against potential limitations and impairments regarding the surgical field and safety. In all cases in this study, dorsal instrumentation was performed *via* percutaneous pedicle screw placement.

## 5. Conclusions

Navigated lumbar dorsal and lateral instrumentation in a single operation and 45-degree positioning is feasible and safe. It enables rapid 360-degree instrumentation in these critically ill patients and potentially reduces overall intraoperative radiation exposure for patient and staff. Compared to purely dorsal approaches it allows for optimal discectomy and fusion while overall incisions and wound size are minimized. Compared to prone LLIF procedures, semi-prone in 45-degree positioning allows for a steep learning curve due to minor changes of familiar anatomy.

## Data Availability

The raw data supporting the conclusions of this article will be made available by the authors, without undue reservation.
